# Effects of fasting and nutritional restriction on the isotopic ratios of nitrogen and carbon: a meta‐analysis

**DOI:** 10.1002/ece3.1738

**Published:** 2015-10-08

**Authors:** Eric Hertz, Marc Trudel, Marlin K. Cox, Asit Mazumder

**Affiliations:** ^1^ Department of BiologyUniversity of VictoriaPO BOX 3020Station CSCVictoriaBCCanadaV8W 3N5; ^2^ Pacific Biological StationDepartment of Fisheries and Oceans Canada3190 Hammond Bay RoadNanaimoBCCanadaV9T 6N7; ^3^ University of Alaska Southeast11120 Glacier HwyJuneauAlaska99801

**Keywords:** Chinook Salmon, diet, food webs, *Oncorhynchus tshawytscha*, stable isotopes, starvation, trophic

## Abstract

Many organisms experience fasting in their life time, and this physiological process has the potential to alter stable isotope values of organisms, and confound interpretation of food web studies. However, previous studies on the effects of fasting and starvation on stable isotopes show disparate results, and have never been quantitatively synthesized. We performed a laboratory experiment and meta‐analysis to determine how stable isotopes of *δ* ^15^N and *δ* ^13^C change with fasting, and we tested whether moderators such as taxa and tissue explain residual variation. We collected literature data from a wide variety of taxa and tissues. We surveyed over 2000 papers, and of these, 26 met our selection criteria, resulting in 51 data points for *δ* ^15^N, and 43 data points for *δ* ^13^C. We determine that fasting causes an average increase in the isotopic value of organisms of 0.5‰ for *δ* ^15^N and that the only significant moderator is tissue type. We find that the overall effect size for *δ* ^13^C is not significant, but when the significant moderator of tissue is considered, significant increases in blood and whole organisms are seen with fasting. Our results show that across tissues and taxa, the nutritional status of an organism must be considered when interpreting stable isotope data, as fasting can cause large differences in stable isotope values that would be otherwise attributed to other factors.

## Introduction

Stable isotopes have become a common method to study many different aspects of food web ecology. For example, stable isotopes have been used to determine the contribution of different factors to food chain length (Post et al. 2000; Vander Zanden and Rasmussen 2001), to determine how human impacts can affect food web structure (Nam et al. 2011), and to determine how predator–prey mass ratios vary in different systems (Jennings et al. 2002; Jennings and Warr 2003; Hertz et al. 2014). Given isotopic separation between prey sources, the contribution of different diet sources to a consumer, thus where an organism fits into a food web, can also be determined using stable isotope analysis (Parnell et al. 2010). Different stable isotopes can elucidate different aspects of food web structure, with nitrogen (*δ* ^15^N) and carbon (*δ* ^13^C) generally being the most commonly used isotopes. *δ* ^15^N is typically used as an indicator of trophic position (Vander Zanden and Rasmussen 2001) with the difference between an organism and its diet usually assumed to be 3.4‰ (Post 2002), though the value of this discrimination may decrease with increasing dietary *δ* ^15^N (Hussey et al. 2014). *δ* ^13^C is more conserved in food webs, with the difference between an organism and diet of around 0–1‰, and thus better serves as an indicator of the source of primary production (DeNiro and Epstein 1978; Miller et al. 2008).

In the application of stable isotopes to food web ecology, the assumption is often made that nutritional status (nutritional restriction, fasting, or starvation) has no effect on the isotopic values of an organism. Yet, few studies consider these possible effects on isotopic values and if this assumption is not met, the conclusions that we draw from stable isotope studies on food webs could be misleading (e.g., Hobson et al. 1993; Bowes et al. 2014). Furthermore, despite some research on the effects of nutritional status of an organism on stable isotopic values (reviewed in Hatch 2012), there is still little consensus in the literature on how the isotopic values of organisms change with fasting and starvation. Generally during fasting, an organism catabolizes lipid reserves, before switching to catabolize proteins (Doucett et al. 1999; Hatch 2012). *δ* ^15^N values are often seen to increase with fasting once an organism begins to catabolize tissues (Hobson et al. 1993; Gaye‐Siessegger et al. 2004; Bowes et al. 2014). This enrichment in *δ* ^15^N should only occur when fasting or starvation is severe enough to cause protein, rather than lipid, catabolism (Martinez del Rio and Wolf 2005; Hatch 2012). However, possibly because of this threshold effect, other studies report no effects of starvation on *δ* ^15^N values (Milanovich and Maerz 2013), or even a decrease in *δ* ^15^N values (Aguilar et al. 2014). Nutritional stress is also expected to result in enrichment of *δ* ^13^C due to processing of lipid reserves. As lipids are depleted in *δ* ^13^C relative to other tissues (DeNiro and Epstein 1977; McConnaughey and McRoy 1979), metabolism of the lipid pool should result in enrichment of remaining tissues (Tieszen et al. 1983; Doucett et al. 1999). As with studies on *δ* ^15^N, some studies also report no significant changes in *δ* ^13^C with fasting (e.g., Varela et al. 2015). For both *δ* ^15^N and *δ* ^13^C, the effects of fasting and starvation on tissue isotopes may depend on the degree and duration of fasting, and the turnover time of the tissue analyzed (Hatch 2012).

In this study, we performed an experiment to determine how the stable isotope values of multiple tissues of juvenile Chinook Salmon (*Oncorhynchus tshawytscha*) change with food deprivation over a 6‐week period. As there are many contrasting results on the effects of nutritional restriction on the isotopic values of organisms, and these effects could have important implications on the interpretation of food web studies using isotopes, we then performed a meta‐analysis to determine the effect of nutritional restriction on the *δ* ^15^N and *δ* ^13^C values of organisms. We examined possible sources of variation in these studies, including experimental duration, tissue analyzed, organism taxa, and initial isotopic value. Using the results from this meta‐analysis, we then perform a sensitivity analysis to determine how fasting could influence estimates of diet contributions to a consumer in a food web. Overall, we find a significant effect of nutritional restriction on the *δ* ^15^N values of organisms and that tissue type is the only significant moderator of this effect. This change in *δ* ^15^N values due to nutritional status, equivalent to approximately one‐seventh of a trophic level, may have the potential to alter the interpretation of food web studies.

## Methods

### Laboratory experiment

To test the response of multiple tissues of juvenile Chinook Salmon to nutritional restriction, juvenile Chinook Salmon were food‐deprived for a period of 6 weeks from 17 November 2011 to 27 December 2011. The initial size of the juvenile Chinook Salmon was 48.4 g (±27 g SD), spanning a range of sizes of approximately 10–80 g (approximately 100–200 mm fork length). The water temperature was between 7.6 and 9.1°C. At the end of weeks 1, 2, 3, 4 and 6, a total of fifteen salmon were lethally sampled, except for weeks 3 and 6, where eight and six samples were available, respectively.

Juvenile Chinook Salmon were dissected to collect tissues for stable isotope analysis. A piece of dorsal muscle tissue was sampled from just below the dorsal fin. A piece of the liver was also removed. Tissues were freeze‐dried, and ground to a fine powder using a heavy‐duty Wig‐L‐Bug grinder.

The powder was weighed (to a thousandth of a milligram) and packed for analysis via a Thermo Delta IV Isotope Ratio Mass Spectrometer (University of Victoria, Victoria, British Columbia). Samples were analyzed for ^13^C and ^15^N stable isotope ratios (*δ* ^13^C and *δ* ^15^N respectively) and expressed in the standard delta (*δ*) notation

(1) $$\delta^{15}N\;\text{or}\;\delta^{13}C=\left(\frac{R_{\mathrm{sample}}}{R_{\mathrm{standard}}}-1\right)\times 1000$$

where *R* = ^13^C/^12^C or ^15^N/^14^N.

We used the residuals from a length–weight regression to compare how the condition of juvenile Chinook Salmon changed over the course of the experiment. We used general linear models to compare the response of *δ* ^15^N and *δ* ^13^C isotopic values of liver and muscle over the sampling period, while taking into account the effects of size. Week was used as a categorical variable, and weight was continuous. We also tested for an interaction between weight and week for each isotope/tissue combination.

### Meta‐analysis

#### Data sources and study selection criteria

**Figure 1:**
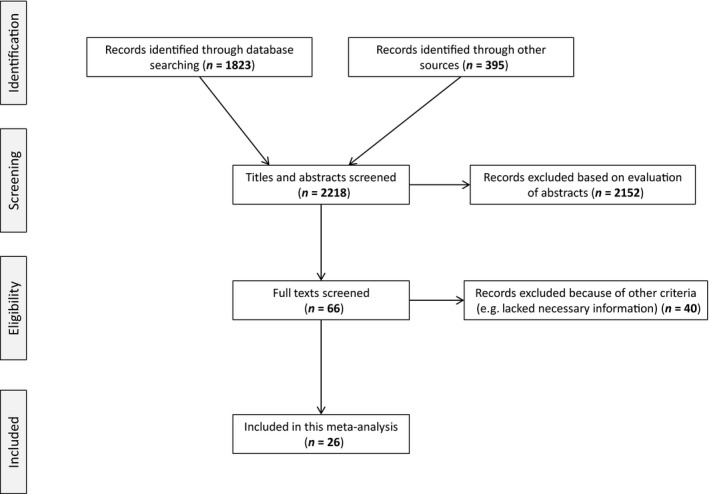
PRISMA diagram for the selection of papers in our meta‐analysis.

To identify primary literature that examined the role of fasting (or diet restriction) on *δ* ^15^N and *δ* ^13^C values of organisms, we systematically searched ISI Web of Science using the following search terms: stable isotop* starv*, stable isotop* nutr* restrict*, and stable isotop* fasting (Fig. 1). We searched the references cited in the review by Lee et al. (2012), and we used Web of Science to search all of the papers that have cited Hobson et al. (1993)—the seminal paper on the effects of starvation on the isotopic values of organisms. Finally, we also included the results from the experiment reported in this study.

We made an initial assessment of relevance based on the title and abstract (Fig. 1). Our search criteria included the following: studies must reduce ration or food‐deprive the organism of interest (e.g., altering nitrogen content of diet not sufficient), the duration of food deprivation must be recorded, and stable isotopes of a control group must be reported. Observational studies that correlate isotopic values of organisms with indices of condition do not meet our search criteria as there is not a control in these situations. In addition, the low number of these types of studies that we found (*n* = 8) precluded us from doing a formal meta‐analysis on these data.

In each paper that met our selection criteria, we extracted sample size, mean, and standard deviation for stable isotope values of control organisms, as well as the organisms at the end of the experimental period. When standard deviation was not reported, we calculated it from the standard error. We extracted all data from tables and text when possible, and used the program DataThief to extract data from figures. We removed all studies that did not report the necessary information for our meta‐analysis. For studies that recorded stable isotope values multiple times over the experiment, we simply used the stable isotope values reported at the end of the experiment. Where studies performed multiple comparisons of species, tissue, or treatment type, we treated each estimate as an independent estimate of effect size (though we included study as a random effect in models).

From all studies, we also recorded the experimental treatment (food‐deprived or restricted diet), whether the organism was endothermic or ectothermic, the tissue type that was analyzed (blood, liver, muscle, plasma, whole, and other including bone, feather, and tail), experimental duration (in days), and body size (in g). For body size, we used the average body weight between the initial and final experimental weight. For the few studies that failed to report body size information, we used the literature to estimate body size. For the *δ* ^13^C meta‐analysis, we also recorded whether lipids were chemically extracted from tissues by the authors, as the lipids tend be depleted in *δ* ^13^C values (McConnaughey and McRoy 1979). Most of the estimates included in our study were derived from laboratory experiments, but we also included a small number of field observations that demonstrated fasting under field conditions (*n* = 6).

#### Effect size calculation

We used two separate metrics of effect size in our meta‐analysis. First, to get an estimate of how large of an effect fasting or nutritional restriction has on the scale that is measured in stable isotope studies (‰), we used the effect size of mean difference. This effect size cannot be weighted, and it is less suitable to compare across study designs, but maintains the biologically meaningful scale of ‰ (Koricheva et al. 2013), thus allowing us to estimate the possible effects of fasting and nutritional restriction on the interpretation of stable isotope studies. Mean difference (md) was calculated by

$$\text{md}={\overset{¯}{Y}}_{1}-{\overset{¯}{Y}}_{2}$$

where *Ȳ*_1_ and *Ȳ*_2_ are the estimated mean isotope values.

Then, to determine the effects of moderators and overall significance of models, we used standardized mean difference (Hedge's *d*). This metric of effect size allows for the weighting of studies by their sample sizes and standard deviations, and allows for the more suitable comparison of studies with different designs and standard deviations. To calculate Hedges' *d*, we used

$$d=\;\frac{{\overset{¯}{Y}}_{1}-{\overset{¯}{Y}}_{2}}{\sqrt{\frac{\left(n_{1}-1\right)s_{1}^{2}\;+\;\left(n_{2}-1\right)s_{2}^{2}}{n_{1}\;+\;n_{2}-2}}}J$$

where *Ȳ*_*1*_ and *Ȳ*_*2*_ are the estimated mean isotope values, corresponding to sample sizes *n*_1_ and *n*_*2*_ with standard deviations of *s*_1_ and *s*_2_. *J* is a correction for small sample sizes that corresponds to

$$J=1-\frac{3}{4(n_{1}+n_{2}-2)-1}$$

The variance for Hedges' *d* is

$$v_{d}=\frac{n_{1}+n_{2}}{n_{1}n_{2}}+\frac{d^{2}}{2(n_{1}+n_{2})}$$

To calculate both effect sizes, we used the Metafor package (Viechtbauer 2010) in R (R Core Team 2013).

We performed our meta‐analyses using restricted maximum likelihood estimation to determine the response of *δ* ^15^N and *δ* ^13^C to fasting and nutritional restriction. We performed separate meta‐analyses for *δ* ^15^N and *δ* ^13^C, and separately calculated overall effect size and 95% confidence intervals, using a linear random‐effects model to account for both random sampling variation and variation in the effect size among studies (Koricheva et al. 2013)

$$T_{i}=\theta_{i}+e_{i};\;e_{i}∼N(0,\sigma^{2})$$

where *T*_*i*_ is the observed effect size in the *i*th study, and *θ*_*i*_ is the unknown corresponding true effect. As we were interested in accounting for heterogeneity in the true effects using moderators, we used a mixed‐effects model:

$$\theta_{i}=\beta_{0}+\beta_{1}X_{1i}+\ldots +\beta_{p}X_{pi}+\varepsilon_{i};\;\varepsilon_{i}∼N(0,\tau^{2})$$

where *X*_*pi*_ is the value of the *p*th moderator for the *i*th study. *τ* ^2^ refers to the amount of variability not accounted for by the moderators in the model. We included study as a random effect in all model formulations.

We used the omnibus test to determine the effect of these moderators on model fits (Table 1). We tested the significance of heterogeneity using Cochrane's Q‐test (Hedges and Olkin 1985).

**Table 1:** Moderators tested for each isotope. Number of cases for all study level moderators are given in parentheses

Variable	*δ* ^15^N	*δ* ^13^C
Study level moderators	Food‐deprived (39) vs. Restricted (12) Ectothermic (25) vs. Endothermic (26)	Food‐deprived (34) vs. Restricted (9) Ectothermic (19) vs. Endothermic (24) Lipid correction (12) vs. not (31)
Biological moderators	Tissue	Tissue
	Body size	Body size
	Duration	Duration
	Initial isotopic value	Initial isotopic value

#### Tests of robustness

Publication bias can occur when the probability of a study being published depends on the statistical significance or direction of a result. We evaluated the robustness of the findings from our meta‐analyses to publication bias using funnel plots and through the calculation of the fail‐safe number (Rosenberg 2005).

#### Simulated effects of fasting and nutritional restriction in food webs

To simulate how fasting could influence the findings of food web studies, we used SIAR (Parnell et al. 2010) to run a simulation study with juvenile Chinook Salmon that were collected off the west coast of Vancouver Island in British Columbia, Canada, from 2000 to 2009 (see Tucker et al. (2011) for sampling details). Juvenile Chinook Salmon generally feed on invertebrates and forage fish (Brodeur 1991; Hertz et al. 2015), so these prey items were used as the end members in the mixing model. Zooplankton were sampled via Bongo tows in the spring and fall of 2000–2009. Juvenile Pacific Herring (*Clupea pallasii*) were sampled in fall 2005 and were used as a representative of the forage fish prey (Hertz et al. 2015).

SIAR was run to determine the source contribution of invertebrates and forage fish to juvenile Chinook Salmon diet (*n* = 555) using the trophic enrichment factors in Post (2002). To simulate fasting, we re‐ran SIAR after changing the isotopic values of each juvenile Chinook Salmon to reflect the changes to the isotopes that were observed in this study.

## Results

### Laboratory experiment

**Figure 2:**
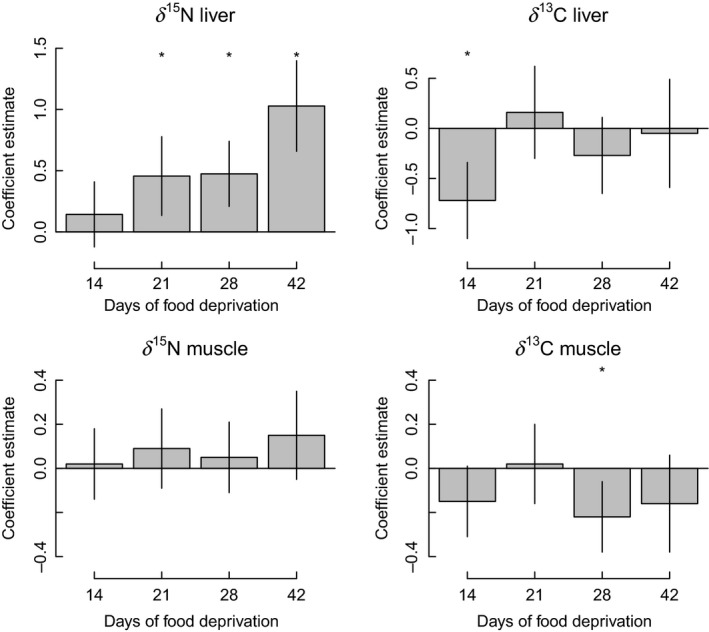
Coefficient estimates for the effect of the number of days since food deprivation was initiated for juvenile Chinook Salmon using a generalized linear model. All estimates are relative to samples taken after 7 days of food deprivation, and estimates account for size differences among fish. Vertical lines represent ± 1 standard deviation. A * refers to a significant difference between the coefficient estimate at that week and the estimate after 7 days.

Juvenile Chinook Salmon lost an average of 5% of their weight over the course of the experiment, and residuals of the length–weight regression went from positive in the first 3 sampling dates (7 days: 3.7 ± 4.9 (mean ± standard deviation); 14 days: 0.7 ± 6.0; 21 days: 0.9 ± 3.9)) to negative in the final two (28 days: −4.0 ± 5.1; 42 days: −2.4 ± 5.0). The final size of the juvenile Chinook Salmon was 46.2 g (±29 g SD). The *δ* ^15^N value of liver and muscle showed a general positive increase over the experimental period (Fig. 2). The linear model for *δ* ^15^N liver showed that there were significant differences between weeks (overall model: *F*(5,53) = 8.4, *P* < 0.0001), with a significant positive coefficient for weight (Fig. 2). For *δ* ^15^N muscle, no week was significantly different from the first week, but weight was a significant predictor (overall model: *F*(5,53) = 16.3, *P* < 0.0001). A general linear model for *δ* ^13^C revealed a significant decrease in values after 14 days for liver, with a significant overall influence of weight (overall model: *F*(5,53) = 16.9, *P* < 0.0001) (Fig. 2). The general linear model for *δ* ^13^C muscle showed a significant decrease in values after 28 days, but not after 42 days (overall model: *F*(5,53) = 3.1, *P* = 0.0171), and weight was not significant There were no significant interactions between week and weight for any of the tissue/isotope combinations (*P* > 0.05).

### Meta‐analysis

#### Systematic literature review

Of the 2218 papers identified in our search, only 26 papers met our selection criteria (Fig. 1). As many studies report changes in both isotopes, there is a considerable overlap in the papers for each meta‐analysis (Table S1). In total for *δ* ^15^N, there were 51 data points from 25 papers. For *δ* ^13^C, there were 43 data points from 22 papers.

For *δ* ^15^N, 76.4% of data points (39 of the 51) came from food‐deprived organisms. The primary taxa group studied was birds (*n* = 17), followed by nine data points from each of mammals, fish, and other (amphibians, coral, planaria, and molluscs), and finally arthropods (*n* = 7) (Table 2). There was a nearly even split between ectothermic and endothermic organisms, with endotherms making up 51% of the data points. The primary tissue analyzed was whole organism (*n* = 13), followed by blood (*n* = 12), other (e.g., bone, milk, feather; *n* = 9), plasma (*n* = 7), muscle (*n* = 6), and finally liver (*n* = 4). The duration of experiment ranged from 4 to 243 days with a mean of 54.9 days. The initial *δ* ^15^N values ranged from 0.3‰ to 19.2‰, with a mean of 9.7‰.

**Table 2:** Contingency table of sample sizes for each tissue type by body size and ‐thermy combinations for *δ* ^15^N

	Blood	Liver	Muscle	Other	Plasma	Whole	Total
(a) Body size							
L (1–4000 kg)	7	0	1	1	7	0	16
M (1 g–1 kg)	5	4	4	5	0	2	20
S (0–1 g)	0	0	1	3	0	11	15
Total	12	4	6	9	7	13	51
(b) –thermy							
Ectothermic	1	3	4	4	0	13	25
Endothermic	11	1	2	5	7	0	26
Total	12	4	6	9	7	13	51

For *δ* ^13^C, 34 of 43 (79%) of the data points came from organisms that were food‐deprived rather than ration‐restricted. Birds were the predominant taxa group (*n* = 15), followed by mammals (*n* = 9), arthropods (*n* = 7), and fish and other (*n* = 6) (Table 2). 56% (24 of the 43) data points came from endothermic organisms. The sample was chemically lipid‐corrected by the authors in only 28% of the data points. The tissue analyzed for *δ* ^13^C was similar to that of *δ* ^15^N, with most being whole organism (*n* = 13), blood (*n* = 12), other (*n* = 11), and plasma (*n* = 7). For *δ* ^13^C, we included liver and muscle in the “other” category as there were only three data points in each of these categories. The duration of experiments ranged from 4 to 243 days with a mean of 58.7 days. The range of mean control *δ* ^13^C values was −26.6‰ to −15.7‰ (mean: −20.7‰).

#### *δ* ^15^N model

**Figure 3:**
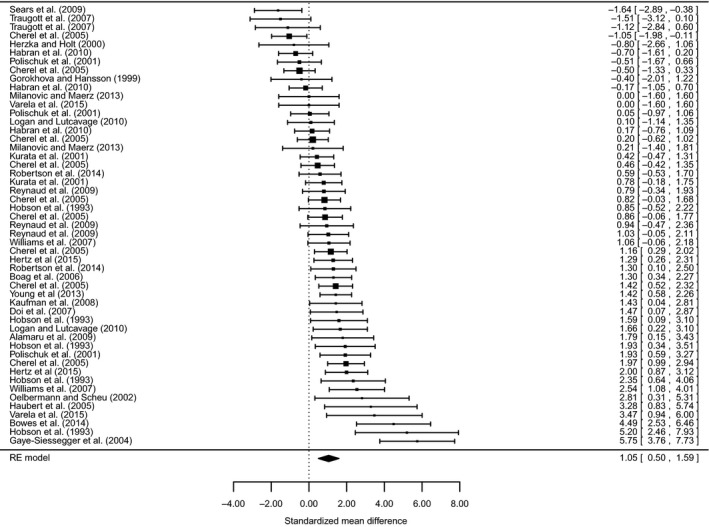
Forest plot of weighted effect sizes (standardized mean differences) with 95% confidence intervals for studies that report data on the *δ* ^15^N of organisms that were food‐deprived or had significantly reduced rations. Confidence intervals that overlap the dashed vertical line at zero are not significant.

The meta‐analysis using the mean difference as the effect size showed that fasting and nutritional restriction result in a significant average increase for *δ* ^15^N of 0.5‰ (95% CI: 0.26–0.74; *n* = 51). The overall weighted mean effect size for the *δ* ^15^N random‐effects meta‐analysis using the standardized mean difference (Hedges' *d*) was 1.05 (95% CI: 0.50–1.59; *n* = 51) (Fig. 3). This indicates a significant positive effect of fasting and nutritional restriction on *δ* ^15^N values across studies. The overall heterogeneity was *Q *=* *181.3 (*P* < 0.0001), indicating that there was significant unaccounted‐for variation between experiments.

Neither fasting versus nutritional restriction (*Q*_M_ = 2.9, *P* = 0.09) nor endothermic versus ectothermic (*Q*_M_ = 1.3, *P* = 0.26) accounted for a significant level of variation. Similarly, body size (*Q*_M_ = 1.2, *P* = 0.27), duration (*Q*_M_ = 0.12, *P* = 0.73), and mean control *δ* ^15^N (*Q*_M_ = 0.65, *P* = 0.42) were not significant moderators. Tissue significantly influenced *δ* ^15^N values (*Q*_M_ = 11.4, *P* = 0.04), suggesting that different tissues may differ in their magnitude of response to nutritional restriction. The model that included tissue as a moderator had residual heterogeneity (*Q *=* *157.1, *P* < 0.0001). Blood, liver, and whole organisms all showed significantly larger effect sizes than 0, while the 95% confidence intervals for muscle, plasma, and other all overlapped with 0 (Fig. 5).

#### *δ* ^13^C model

**Figure 4:**
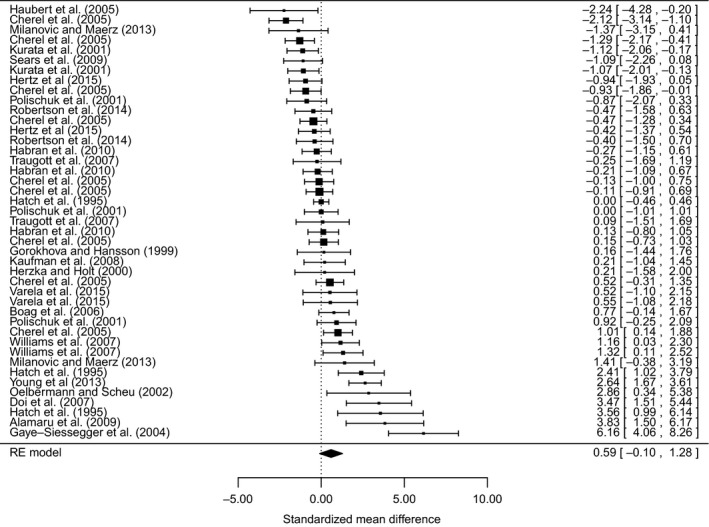
Forest plot of weighted effect sizes (standardized mean differences) with 95% confidence intervals for studies that report data on the *δ* ^13^C of organisms that were food‐deprived or had significantly reduced rations. Confidence intervals that overlap the dashed vertical line at zero are not significant.

**Figure 5:**
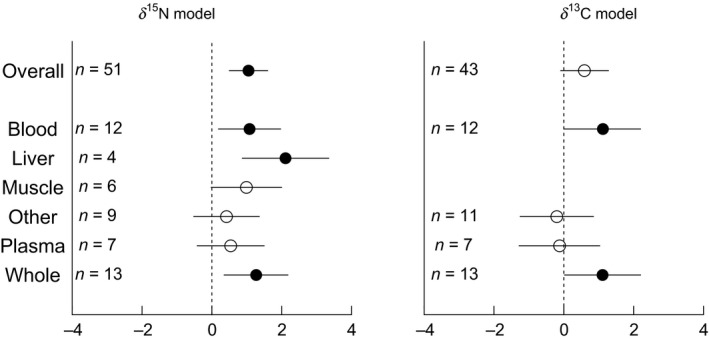
Tissue‐specific responses of the effect sizes (with 95% confidence intervals) from *δ* ^15^N and *δ* ^13^C random‐effects models. Effect sizes that are significantly different from zero are shown in filled circles, while confidence intervals that overlap the dashed vertical line at zero are not significant (open circles).

For *δ* ^13^C, the meta‐analysis using the effect size of mean difference showed that fasting and nutritional restriction result in a non‐significant increase of 0.31‰ (95% CI: 0.003–0.62; *n* = 43). For the *δ* ^13^C random‐effects meta‐analysis, the overall weighted mean effect size was 0.59 (95% CI: −0.10–1.28; *n* = 43), indicating that there was no consistent effect of fasting and nutritional restriction on *δ* ^13^C values across studies (Fig. 4). There was significant unaccounted‐for variation between studies, with an overall heterogeneity of *Q *=* *190.4 (*P* < 0.0001). Tissue was again the only significant moderator (*Q*_M_ **=* *26.3, *P* = 0.001) (Fig. 5), and there was significant heterogeneity remaining in this model (*Q *=* *173.4, *P* < 0.0001). Similar to the *δ* ^15^N model, blood and whole organism had significantly larger effect sizes than 0, while plasma and other did not (Fig. 5). None of fasting versus nutritional restriction (*Q*_M_ **=* *0.33, *P* = 0.56), endotherm versus ectotherm (*Q*_M_ **=* *0.53, *P* = 0.48), or lipid extraction (*Q*_M_ **=* *0.10, *P* = 0.76) were significant moderators. Similarly, body size (*Q*_M_ **=* *0.30, *P* = 0.59), duration (*Q*_M_ **=* *2.3, *P* = 0.07), and mean control *δ* ^13^C (*Q*_M_ **=* *0.07, *P* = 0.79) were not significant moderators.

#### Robustness

For the overall *δ* ^15^N model, the fail‐safe number was 1819, meaning that this number of non‐significant studies would have to be added to our data set to change the significance of the result. The funnel plot was largely symmetrical (Fig. S1), suggesting that publication bias did not significantly bias our results. For the overall *δ* ^13^C model, the fail‐safe number was much lower (25), though the fail‐safe number for an insignificant result is not meaningful (Côté and Sutherland 1997). The funnel plot for *δ* ^13^C was also largely symmetrical (Fig. S2).

#### Simulated effects of fasting and nutritional restriction in food webs

**Figure 6:**
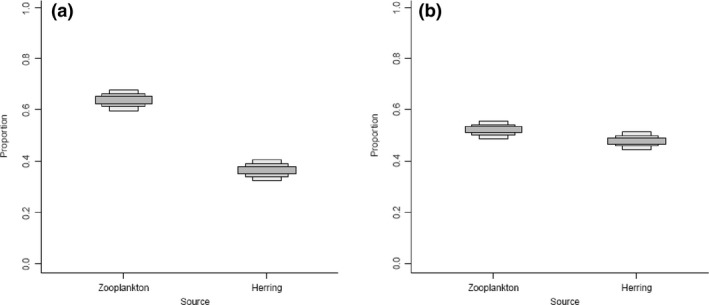
Credible interval plot from SIAR of the contribution of different diet sources to juvenile Chinook Salmon muscle tissue. Intervals go from 50%, 75%, and 95% credible intervals from dark to light gray boxes. (A) is the results from the sampled fish, while (B) is the simulated results with each juvenile Chinook Salmon having *δ* ^15^N values increased by the results found in this study (0.50‰).

Simulated fasting changed the magnitude of the contribution of different prey sources to juvenile Chinook Salmon (Fig. 6). The simulated fast resulted in an increase in the proportion of diet from Pacific Herring (Fig. 6B), resulting in a more even distribution of prey sources than in reality (Fig. 6A).

## Discussion

By making a number of simplifying assumptions, researchers have been able to use stable isotopes to study a wide variety of food web questions. The most common two assumptions to be considered are that (1) the discrimination value is known and constant, and (2) the organism is at equilibrium with their diet. The sensitivity of many food web studies is beginning to be tested with respect to these assumptions. Here, we show that another tacit assumption in stable isotopes studies, that nutritional status of an organism does not affect stable isotope values, must also be considered. We find that fasting and reduced ration caused a significant increase in *δ* ^15^N values, and tissue‐specific overall responses in *δ* ^13^C values. Over the duration of the experiments, we found that *δ* ^15^N values became enriched by an average of 0.5‰—a possibly significant amount in food web studies.

These findings have implications for the interpretation of food web studies. If an organism under study is undergoing fasting or nutritional restriction, values of *δ* ^15^N (and possibly *δ* ^13^C) will become enriched. If nutritional status is not considered, it could appear that the organism has experienced a trophic shift or is feeding on a different resource, when, in reality, it is catabolizing its own tissues. For example, Welch and Parsons (1993) measured stable isotopes in the carcasses of Sockeye Salmon (*Oncorhynchus nerka*) from five different populations after they had completed their spawning migration. They showed that the carbon isotope ratios of these different populations were fairly similar. However, the *δ* ^15^N differed by 1–2‰ among populations. Welch and Parsons (1993) argued that these differences were likely due to differences in the spatial distribution of these populations in the open ocean, but these differences could also be related to fasting. Adult Sockeye Salmon stop feeding during their upstream migration and rely on the energy reserves accumulated during their marine life to fuel their metabolic functions. Lipids are the primary source of energy they use during their migration, but proteins can also be catabolized (Hendry and Berg 1999). The quantity of lipids stored prior to the upstream migration varies among populations and appears to be related to the migration difficulty (Crossin et al. 2004). Different populations may therefore be catabolizing a different proportion of their proteins during their migration until they reach senescence. Hence, part of the differences observed in *δ* ^15^N values among populations could be due to the physiological changes associated with fasting and migration itself. Alternatively, some of the enrichment in *δ* ^15^N values within Sockeye Salmon could be the result of morphological changes, as migrating male Sockeye Salmon also build new structural tissue associated with mating, which could also result in enrichment of *δ* ^15^N (Tibbets et al. 2008; Martinez del Rio et al. 2009).

### *δ* ^15^N and *δ* ^13^C models

Hobson et al. (1993) were the first authors to study ecological stable isotopes in the context of starvation. These authors found enrichment of *δ* ^15^N values due to starvation, as the birds under study were essentially consuming their own tissues as they were starved, and their tissue became more enriched. Subsequent studies have found somewhat mixed results, however, by synthesizing all studies in a meta‐analytic framework, and we find a significant overall effect size for *δ* ^15^N.

Possibly because of the wide range of taxa, tissue, duration, and experimental designs included in this meta‐analysis, we found that many moderators did not significantly change the effect size of the *δ* ^15^N and *δ* ^13^C models. Most of the moderators that we tested were, in some way, related to tissue turnover rates. For example, duration of the study is only really meaningful when considered with respect to the turnover rate (Logan and Lutcavage 2010). Turnover rate, in turn, depends on tissue, taxa, and body size (Vander Zanden et al. 2015). It is possible that interactions between our moderators were, in fact, responsible for the difference in effect sizes between studies, but we did not have the sample sizes available to study these interactions (Table 2). As more studies become available, the linkages between these variables will become easier to study (e.g., Vander Zanden et al. 2015).

Of the moderators that we tested for both *δ* ^15^N and *δ* ^13^C, we found that tissue was the only significant one. We found that tissues responded in varying degrees to fasting and nutritional restriction. The relative turnover time was not indicative of response to fasting, with some relatively faster‐response tissues showing significant (liver) and insignificant responses (plasma). This variability could be because these tissues were from a collection of taxa over varying durations.

### Limitations and future directions

During our literature review, we had to eliminate a number of studies because they did not report all of the necessary information that was required to calculate effect sizes. Despite this, the high value of our fail‐safe number and symmetry in the funnel plots indicate that the *δ* ^15^N model would be robust to the inclusion of more of these studies. The exclusion of these studies may have had more of an effect on the *δ* ^13^C model, where the overall effect size was insignificant, but only barely so.

While our meta‐analysis was able to show that tissue‐ and isotope‐specific responses to fasting, careful experimentation is required for to understand the processes underlying this pattern. Experiments are need to (1) better quantify the effects of fasting on isotopic turnover, (2) understand the mechanisms underlying fractionation associated with fasting, and (3) determine which processes are discriminating and which are not.

## Conclusions

As stable isotopes become an increasing method in the toolbox of ecologists, the assumptions underlying this analysis must become more rigorously tested. Here, we used a meta‐analysis to show the significant effects of fasting and nutritional restriction on the isotope ratios in animal tissues. We thus suggest that researchers should consider nutritional status in the interpretation of stable isotope studies.

## Conflict of Interest

None declared.

## Supporting information


**Figure S1.** Funnel plot for δ^15^N meta‐analysis model.
**Figure S2.** Funnel plot for δ^13^C meta‐analysis model.
**Table S1.** Description of papers and moderators used in the meta‐analysis.
