# Supplementary material for: Effects of fasting and nutritional restriction on the isotopic ratios of nitrogen and carbon: a meta‐analysis
Source: Ecol Evol. 2015 Oct 8;5(21):4829–39. doi: 10.1002/ece3.1738 (PMC4662305; doi:10.1002/ece3.1738)
Supplement: Supplementary file 1 — Figure S1. Funnel plot for δ15N meta‐analysis model. Figure S2. Funnel plot for δ13C meta‐analysis model. Table S1. Description of papers and moderators used in the meta‐analysis. [file ECE3-5-4829-s001.docx]

**Supporting Information:**

Table S1: Description of papers and moderators used in the meta-analysis

| \| Paper \| Species \| Sp \| Weight \| Treatment \| Duration \| Therm \| Taxa \| Tissue \| Lipid \| \| --- \| --- \| --- \| --- \| --- \| --- \| --- \| --- \| --- \| --- \| \| Alamaru et al. (2009) \| Hood coral \| Stylophora pistillata \| 0.0001 \| Starvation \| 14 \| Ecto \| Other \| Whole \| No \| \| Boag et al. (2006) \| New Zealand Flatworm \| Arthurdendyus triangulatus \| 0.5 \| Starvation \| 243 \| Ecto \| Other \| Whole \| No \| \| Bowes et al. (2014) \| Freshwater guppy \| Poecilia reticulata \| 0.35 \| Restricted \| 120 \| Ecto \| Fish \| Muscle \|  \| \| Cherel et al. (2005) \| King Penguin \| Aptenodytes patagonicus \| 12885 \| Starvation \| 21 \| Endo \| Bird \| Blood \| No \| \| Cherel et al. (2005) \| King Penguin \| Aptenodytes patagonicus \| 12885 \| Starvation \| 21 \| Endo \| Bird \| Blood \| No \| \| Cherel et al. (2005) \| King Penguin \| Aptenodytes patagonicus \| 12885 \| Starvation \| 21 \| Endo \| Bird \| Plasma \| No \| \| Cherel et al. (2005) \| King Penguin \| Aptenodytes patagonicus \| 12425 \| Starvation \| 25 \| Endo \| Bird \| Blood \| No \| \| Cherel et al. (2005) \| King Penguin \| Aptenodytes patagonicus \| 12425 \| Starvation \| 25 \| Endo \| Bird \| Blood \| No \| \| Cherel et al. (2005) \| King Penguin \| Aptenodytes patagonicus \| 12425 \| Starvation \| 25 \| Endo \| Bird \| Plasma \| No \| \| Cherel et al. (2005) \| King Penguin \| Aptenodytes patagonicus \| 7295 \| Starvation \| 120 \| Endo \| Bird \| Blood \| No \| \| Cherel et al. (2005) \| King Penguin \| Aptenodytes patagonicus \| 7295 \| Starvation \| 120 \| Endo \| Bird \| Blood \| No \| \| Cherel et al. (2005) \| King Penguin \| Aptenodytes patagonicus \| 7295 \| Starvation \| 120 \| Endo \| Bird \| Plasma \| No \| \| Doi et al. (2007) \| Chironomid larvae \| Chironomus acerbiphilus \| 0.185 \| Starvation \| 12 \| Ecto \| Arthropod \| Whole \| No \| \| Gaye-Siessegger et al. (2004) \| Carp \| Cyprinus carpio \| 29.25 \| Restricted \| 56 \| Ecto \| Fish \| Whole \| Yes \| \| Gorokhova and Hansson (1999) \| Mysid \| Mysis mixta \| 1.25 \| Starvation \| 35 \| Ecto \| Arthropod \| Whole \| No \| \| Habran et al. (2010) \| Elephant seal \| Mirounga angustirostris \| 391500 \| Starvation \| 17 \| Endo \| Mammal \| Blood \| No \| \| Habran et al. (2010) \| Elephant seal \| Mirounga angustirostris \| 391500 \| Starvation \| 17 \| Endo \| Mammal \| Other \| No \| \| Habran et al. (2010) \| Elephant seal \| Mirounga angustirostris \| 391500 \| Starvation \| 17 \| Endo \| Mammal \| Plasma \| No \| \| Hatch et al. (1995) \| Chicken \| Gallus gallus domesticus \| 84 \| Restricted \| 12 \| Endo \| Bird \| Blood \| No \| \| Hatch et al. (1995) \| Chicken \| Gallus gallus domesticus \| 84 \| Restricted \| 12 \| Endo \| Bird \| Other \| No \| \| Hatch et al. (1995) \| Chicken \| Gallus gallus domesticus \| 79 \| Restricted \| 43 \| Endo \| Bird \| Blood \| No \| \| Haubert et al. (2005) \| Collembola \| Protaphorura fimata \| 0.00125 \| Starvation \| 28 \| Ecto \| Arthropod \| Whole \| No \| \| Hertz et al (2015) \| Chinook salmon \| Oncorhynchus tshawytscha \| 50 \| Starvation \| 35 \| Ecto \| Fish \| Muscle \| No \| \| Hertz et al (2015) \| Chinook salmon \| Oncorhynchus tshawytscha \| 50 \| Starvation \| 35 \| Ecto \| Fish \| Liver \| No \| \| Herzka and Holt (2000) \| Red drum \| Sciaenops ocellatus \| 0.0006 \| Starvation \| 4 \| Ecto \| Fish \| Whole \| No \| \| Hobson et al. (1993) \| Quail \| Coturnix japonica \| 40 \| Restricted \| 18 \| Endo \| Bird \| Blood \|  \| \| Hobson et al. (1993) \| Quail \| Coturnix japonica \| 40 \| Restricted \| 18 \| Endo \| Bird \| Muscle \|  \| \| Hobson et al. (1993) \| Quail \| Coturnix japonica \| 40 \| Restricted \| 18 \| Endo \| Bird \| Liver \|  \| \| Hobson et al. (1993) \| Quail \| Coturnix japonica \| 40 \| Restricted \| 18 \| Endo \| Bird \| Other \|  \| \| Hobson et al. (1993) \| Quail \| Coturnix japonica \| 40 \| Restricted \| 18 \| Endo \| Bird \| Other \|  \| \| Kaufman et al. (2008) \| Amphipod \| Onisimus litoralis \| 0.0579 \| Starvation \| 63 \| Ecto \| Arthropod \| Whole \| No \| \| Kurata et al. (2001) \| Salt marsh snail \| Assiminea japonica \| 1 \| Starvation \| 30 \| Ecto \| Other \| Whole \| Yes \| \| Kurata et al. (2001) \| Salt marsh snail \| Angustasiminea castanea \| 1 \| Starvation \| 30 \| Ecto \| Other \| Whole \| Yes \| \| Logan and Lutcavage (2010) \| Coastal skate \| Leucoraja spp. \| 800 \| Starvation \| 20 \| Ecto \| Fish \| Blood \|  \| \| Logan and Lutcavage (2010) \| Coastal skate \| Leucoraja spp. \| 800 \| Starvation \| 20 \| Ecto \| Fish \| Muscle \|  \| \| Milanovic et al. (2014) \| Red backed salamader \| Plethodon cinereu \| 124.35 \| Starvation \| 35 \| Ecto \| Other \| Liver \| No \| \| Milanovic et al. (2014) \| Red backed salamader \| Plethodon cinereu \| 124.35 \| Starvation \| 35 \| Ecto \| Other \| Other \| No \| \| Oelbermann and Scheu (2002) \| Spider \| Pardosa lugubris \| 0.0006 \| Starvation \| 12 \| Ecto \| Arthropod \| Whole \| No \| \| Polischuk et al. (2001) \| Polar bear \| Ursus maritimus \| 260000 \| Starvation \| 105 \| Endo \| Mammal \| Plasma \| Yes \| \| Polischuk et al. (2001) \| Polar bear \| Ursus maritimus \| 260000 \| Starvation \| 105 \| Endo \| Mammal \| Plasma \| Yes \| \| Polischuk et al. (2001) \| Polar bear \| Ursus maritimus \| 260000 \| Starvation \| 225 \| Endo \| Mammal \| Plasma \| Yes \| \| Reynaud et al. (2009) \| Hood coral \| Stylophora pistillata \| 0.0001 \| Starvation \| 63 \| Ecto \| Other \| Other \|  \| \| Reynaud et al. (2009) \| Hood coral \| Stylophora pistillata \| 0.0001 \| Starvation \| 63 \| Ecto \| Other \| Other \|  \| \| Reynaud et al. (2009) \| Hood coral \| Stylophora pistillata \| 0.0001 \| Starvation \| 63 \| Ecto \| Other \| Other \|  \| \| Robertson et al. (2014) \| Rat \| Rattus norvegicus \| 260 \| Restricted \| 90 \| Endo \| Mammal \| Other \| Yes \| \| Robertson et al. (2014) \| Rat \| Rattus norvegicus \| 260 \| Restricted \| 180 \| Endo \| Mammal \| Other \| Yes \| \| Sears et al. (2009) \| Rhinocerous auklet \| Cerorhinca monocerata \| 300 \| Restricted \| 34 \| Endo \| Bird \| Blood \| Yes \| \| Traugott et al. (2007) \| Beetle larvae \| Agriotes obscurus \| 0.05 \| Starvation \| 128 \| Ecto \| Arthropod \| Whole \| No \| \| Traugott et al. (2007) \| Beetle larvae \| Agriotes obscurus \| 0.05 \| Starvation \| 128 \| Ecto \| Arthropod \| Whole \| No \| \| Varela et al. (2013) \| Atlantic Bonito \| Sarda sarda \| 445 \| Starvation \| 45 \| Ecto \| Fish \| Muscle \| Yes \| \| Varela et al. (2013) \| Atlantic Bonito \| Sarda sarda \| 445 \| Starvation \| 45 \| Ecto \| Fish \| Liver \| Yes \| \| Williams et al. (2007) \| Tufted puffins \| Fratercula cirrhata \| 400 \| Restricted \| 27 \| Endo \| Bird \| Blood \| No \| \| Williams et al. (2007) \| Tufted puffins \| Fratercula cirrhata \| 400 \| Restricted \| 68 \| Endo \| Bird \| Blood \| No \| \| Young et al (2013) \| Ringed seal \| Phoca hispida \| 60000 \| Starvation \| 60 \| Endo \| Mammal \| Muscle \| Yes \| |  |  |  |  |  |  |  |  |  |  |  |
| --- | --- | --- | --- | --- | --- | --- | --- | --- | --- | --- | --- | --- | --- | --- | --- | --- | --- | --- | --- | --- | --- | --- | --- | --- | --- | --- | --- | --- | --- | --- | --- | --- | --- | --- | --- | --- | --- | --- | --- | --- | --- | --- | --- | --- | --- | --- | --- | --- | --- | --- | --- | --- | --- | --- | --- | --- | --- | --- | --- | --- | --- | --- | --- | --- | --- | --- | --- | --- | --- | --- | --- | --- | --- | --- | --- | --- | --- | --- | --- | --- | --- | --- | --- | --- | --- | --- | --- | --- | --- | --- | --- | --- | --- | --- | --- | --- | --- | --- | --- | --- | --- | --- | --- | --- | --- | --- | --- | --- | --- | --- | --- | --- | --- | --- | --- | --- | --- | --- | --- | --- | --- | --- | --- | --- | --- | --- | --- | --- | --- | --- | --- | --- | --- | --- | --- | --- | --- | --- | --- | --- | --- | --- | --- | --- | --- | --- | --- | --- | --- | --- | --- | --- | --- | --- | --- | --- | --- | --- | --- | --- | --- | --- | --- | --- | --- | --- | --- | --- | --- | --- | --- | --- | --- | --- | --- | --- | --- | --- | --- | --- | --- | --- | --- | --- | --- | --- | --- | --- | --- | --- | --- | --- | --- | --- | --- | --- | --- | --- | --- | --- | --- | --- | --- | --- | --- | --- | --- | --- | --- | --- | --- | --- | --- | --- | --- | --- | --- | --- | --- | --- | --- | --- | --- | --- | --- | --- | --- | --- | --- | --- | --- | --- | --- | --- | --- | --- | --- | --- | --- | --- | --- | --- | --- | --- | --- | --- | --- | --- | --- | --- | --- | --- | --- | --- | --- | --- | --- | --- | --- | --- | --- | --- | --- | --- | --- | --- | --- | --- | --- | --- | --- | --- | --- | --- | --- | --- | --- | --- | --- | --- | --- | --- | --- | --- | --- | --- | --- | --- | --- | --- | --- | --- | --- | --- | --- | --- | --- | --- | --- | --- | --- | --- | --- | --- | --- | --- | --- | --- | --- | --- | --- | --- | --- | --- | --- | --- | --- | --- | --- | --- | --- | --- | --- | --- | --- | --- | --- | --- | --- | --- | --- | --- | --- | --- | --- | --- | --- | --- | --- | --- | --- | --- | --- | --- | --- | --- | --- | --- | --- | --- | --- | --- | --- | --- | --- | --- | --- | --- | --- | --- | --- | --- | --- | --- | --- | --- | --- | --- | --- | --- | --- | --- | --- | --- | --- | --- | --- | --- | --- | --- | --- | --- | --- | --- | --- | --- | --- | --- | --- | --- | --- | --- | --- | --- | --- | --- | --- | --- | --- | --- | --- | --- | --- | --- | --- | --- | --- | --- | --- | --- | --- | --- | --- | --- | --- | --- | --- | --- | --- | --- | --- | --- | --- | --- | --- | --- | --- | --- | --- | --- | --- | --- | --- | --- | --- | --- | --- | --- | --- | --- | --- | --- | --- | --- | --- | --- | --- | --- | --- | --- | --- | --- | --- | --- | --- | --- | --- | --- | --- | --- | --- | --- | --- | --- | --- | --- | --- | --- | --- | --- | --- | --- | --- | --- | --- | --- | --- | --- | --- | --- | --- | --- | --- | --- | --- | --- | --- | --- | --- | --- | --- | --- | --- | --- | --- | --- | --- | --- | --- | --- | --- | --- | --- | --- | --- | --- | --- | --- | --- | --- | --- | --- | --- | --- | --- | --- | --- | --- | --- | --- | --- | --- | --- | --- | --- | --- | --- | --- | --- | --- | --- | --- | --- | --- | --- | --- | --- | --- | --- | --- | --- | --- | --- | --- | --- | --- | --- | --- | --- | --- | --- | --- | --- | --- | --- | --- | --- | --- | --- | --- | --- |

**References in Supporting Information A:**

Alamaru, A., Yam, R., Shemesh, A. & Loya, Y. (2009) Trophic biology of *Stylophora pistillata* larvae: evidence from stable isotope analysis. *Marine Ecology Progress Series.*, 383, 85-94.

Boag, B., Neilson, R. & Scrimgeour, C. M. (2006) The effect of starvation on the planarian *Arthurdendyus triangulatus* (Tricladida: Terricola) as measured by stable isotopes. *Biolog of Fertile Soils*, 43, 267-270.

Bowes, R.E., Lafferty, M.H. & Thorp J.H. (2014) Less means more: nutrient stress leads to higher δ^15^N ratios in fish. *Freshwater Biology*, 59, 1926-1931.

Cherel, Y., Hobson, K. A., Bailleul, F. & Groscolas, R. (2005) Nutrition, physiology, and stable isotopes: new information from fasting and molting penguins. *Ecology*, 86, 2881-2888.

Doi, H., Kikuchi, E., Takagi, S. & Shikano, S. (2007) Changes in carbon and nitrogen stable isotopes of chironomid larvae during growth, starvation and metamorphosis. *Rapid Communications in Mass Spectrometry,* 21, 997-1002.

Gaye-Siessegger, J., Focken, U., Muetzel, S., Abel, H. & Becker, K. (2004) Feeding level and individual metabolic rate affect δ^13^C and δ^15^N values in carp: implications for food web studies. *Oecologia*, 138, 175-183.

Gorokhova, E. & Hansson, S. (1999) An experimental study on variations in stable carbon and nitrogen isotope fractionation during growth of Mysis mixta and Neomysis integer. *Canadian Journal of Fisheries and Aquatic Sciences,* 56, 2203-2210.

Habran, S., Debier, C., Crocker, D. E., Houser, D. S., Lepoint, G., Bouquegneau, J. M. & Das, K. (2010) Assessment of gestation, lactation and fasting on stable isotope ratios in northern elephant seals (*Mirounga angustirostris*). *Marine Mammal Science,* 26, 880-895.

Hatch, K. A., Sacksteder, K. A., Treichel, I. W., Cook, M. E. & Porter, W. P. (1995) Early detection of catabolic state via change in 13C/12C ratios of blood proteins. *Biochemical and Biophysical Research Communications,* 212, 719-726.

Haubert, D., Langel, R., Scheu, S. & Ruess, L. (2005) Effects of food quality, starvation and life stage on stable isotope fractionation in Collembola. *Pedobiologia*, 49, 229-237.

Herzka, S. Z. & Holt, G. J. (2000) Changes in isotopic composition of red drum (Sciaenops ocellatus) larvae in response to dietary shifts: potential applications to settlement studies. *Canadian Journal of Fisheries and Aquatic Sciences,* 57, 137-147.

Hobson, K.A., Alisauskas, R.T. & Clark, R.G. (1993) Stable-nitrogen isotope enrichment in avian tissues due to fasting and nutritional stress: implications for isotopic analyses of diet. *Condor*, 95, 388-394.

Kaufman, M. R., Gradinger, R. R., Bluhm, B. A. & O’Brien, D. M. (2008) Using stable isotopes to assess carbon and nitrogen turnover in the Arctic sympagic amphipod *Onisimus litoralis*. *Oecologia*, 158, 11-22.

Kurata, K., Minami, H. & Kikuchi, E. (2001) Stable isotope analysis of food sources for salt marsh snails. *Marine Ecology Progress Series*, 223, 167-177.

Logan, J.M. & Lutcavage, M.E. (2010) Stable isotope dynamics in elasmobranch fishes.

*Hydrobiologia*, 644, 231-244.

Milanovich, J.R. & Maerz, J.C. (2013) Realistic fasting does not affect stable isotope levels of a

metabolically efficient salamander. *Journal of Herpetology,* 47, 544-548.

Oelbermann, K. & Scheu, S. (2002) Stable isotope enrichment (δ15N and δ13C) in a generalist predator (*Pardosa lugubris*, Araneae: Lycosidae): effects of prey quality. *Oecologia*, 130, 337-344.

Polischuk, S. C., Hobson, K. A. & Ramsay, M. A. (2001) Use of stable-carbon and-nitrogen isotopes to assess weaning and fasting in female polar bears and their cubs. *Canadian Journal of Zoology,* 79, 499-511.

Reynaud, S., Martinez, P., Houlbrèque, F., Billy, I., Allemand, D. & Ferrier-Pages, C. (2009) Effect of light and feeding on the nitrogen isotopic composition of a zooxanthellate coral: role of nitrogen recycling. *Marine Ecology Progress Series*, 392, 103-110.

Robertson, K. L., Rowland, N. E. & Krigbaum, J. (2014) Effects of caloric restriction on nitrogen and carbon stable isotope ratios in adult rat bone. *Rapid Communications in Mass Spectrometry,* 28, 2065-2074.

Sears, J., Hatch, S. A. & O’Brien, D. M. (2009) Disentangling effects of growth and nutritional status on seabird stable isotope ratios. *Oecologia*, 159, 41-48.

Traugott, M., Pázmándi, C., Kaufmann, R. & Juen, A. (2007) Evaluating 15 N/14 N and 13 C/12 C isotope ratio analysis to investigate trophic relationships of elaterid larvae (Coleoptera: Elateridae). *Soil Biology and Biochemistry,* 39, 1023-1030.

Varela, J.L., Ortega, A., la Gándara, F. & Medina, A. (2013) Effects of starvation on δ^15^N and δ^13^C in Atlantic bonito, *Sarda sarda* (Bloch, 1793). *Aquaculture Research,* 1-5.

Williams, C.T., Buck, C.L., Sears, J. & Kitaysky, A.S. (2007) Effects of nutritional restriction on nitrogen and carbon stable isotopes in growing seabirds. *Oecologia*, 153, 11-18.

Young, B. G. & Ferguson, S. H. (2013) Seasons of the ringed seal: pelagic open-water hyperphagy, benthic feeding over winter and spring fasting during molt. *Wildlife Research,* 40, 52-60.


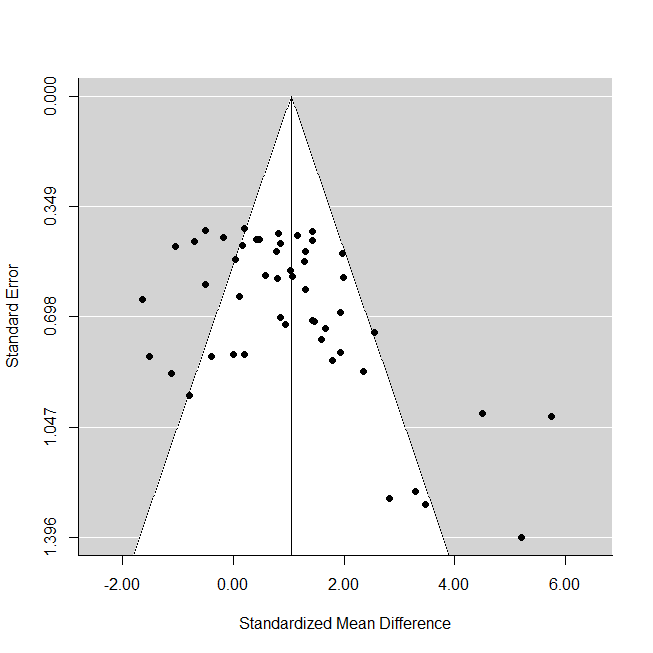


Figure S1: Funnel plot for δ^15^N meta-analysis model


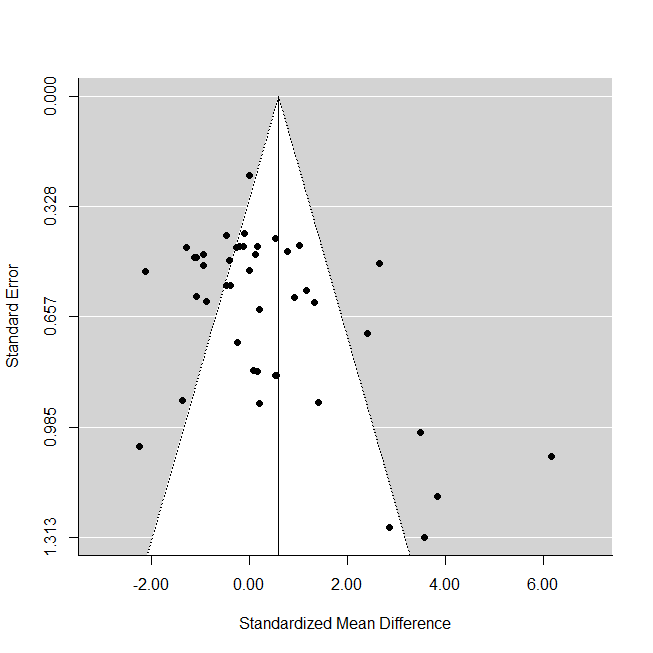


Figure S2: Funnel plot for δ^13^C meta-analysis model
